# Mucosal immunity in health care workers’ respiratory tracts in the post-COVID-19 period

**DOI:** 10.1038/s41598-023-32670-w

**Published:** 2023-05-03

**Authors:** Nadezhda Kryukova, Irina Baranova, Natalia Abramova, Ekaterina Khromova, Dmitry Pachomov, Oksana Svitich, Alexander Chuchalin, Mikhail Kostinov

**Affiliations:** 1 Pirogov Russian National Research Medical University, Moscow, Russian Federation; 2 I. Mechnikov Research Institute of Vaccines and Sera, Moscow, Russian Federation; 3 I.M. Sechenov First Moscow State Medical University, Moscow, Russian Federation

**Keywords:** Immunology, Adaptive immunity, Infection, Mucosal immunology

## Abstract

Coronavirus disease (COVID-19) has generated interest in the assessment of systemic immune status, but existing knowledge about mucosal immunity is clearly insufficient to understand the full pathogenetic mechanisms of the disease. The aim of this study was to evaluate the long-term effects of novel coronavirus infection on mucosal immunity in the postinfection period among health care workers (HCWs). A total of 180 health care workers with and without a history of COVID-19 who ranged in age from 18 to 65 years were enrolled in this one-stage, cross-sectional study. The study subjects completed the 36-Item Short Form (36) Health Survey (SF-36) and the Fatigue Assessment Scale. Secretory immunoglobulin A (sIgA) and total immunoglobulin G (IgG) levels were quantified in saliva samples, induced sputum samples, and nasopharyngeal and oropharyngeal scrapings by an enzyme-linked immunosorbent assay. Specific anti-SARS-CoV-2 IgG antibodies were quantified in serum samples by chemiluminescence immunoassay. Analysis of the questionnaire data showed that all HCWs with a history of COVID-19 reported health problems that limited their daily activities and negative changes in their emotional health three months after the disease, regardless of its severity. The following shifts were detected in the adaptive arm of the immune response in different mucosal compartments. Among subjects who had severe or moderate-to-severe COVID-19, salivary sIgA levels were significantly higher than those in the control group (*p* < 0.05 and *p* < 0.005, respectively). Compared to the subjects in the control group, all subjects with prior COVID-19 had significantly higher levels of total IgG in induced sputum. In the group of patients who had had severe infection, total IgG in saliva was also higher (*p* < 0.05). A direct statistically significant correlation was also detected between the levels of total IgG in all studied samples and the levels of specific IgG antibodies against SARS-CoV-2 in the serum. A significant correlation was observed between total IgG levels and the parameters of physical and social activities, mental health, and fatigue levels. Our study demonstrated long-term changes in the humoral mucosal immune response, which were most pronounced in health care workers with a history of severe or moderate-to-severe COVID-19, and an association of these changes with certain clinical signs of post-COVID-19 syndrome.

## Introduction

Over the past two decades, new viral diseases have emerged and become a major public health threat worldwide. In December 2019, an outbreak of a new highly contagious infection caused by SARS-CoV-2 emerged in Wuhan, China, which subsequently swept the world and took the form of a pandemic. Coronavirus disease 2019 (COVID-19) is caused by SARS-CoV-2, which is characterized by high contagiousness and a severe course. HCWs are at high risk of contracting COVID-19. Our data are based on the results of a survey conducted among doctors and nurses working in one of the hospitals in Moscow.

Clinical manifestations of COVID-19 have been extensively researched. A rich semiology is reported in the literature. In addition to the cardinal signs of COVID-19 (i.e., fever (88–100%), cough (68–85%), and sputum (23–41%)), several other signs have been reported: anorexia (84%), asthenia (70–80%), headache (52–55%), ageusia (43%), anosmia (37%), abdominal pain (25%), neuromuscular involvement (19%), dyspnoea (18–85%), odynophagia (19–20%), myalgia (15–48%), confusion (15%), chest pain (6–20%), stroke (6%), rhinitis (5%), nausea/vomiting (4–5%), diarrhoea (4–15%), and haemoptysis (0–5%)^1–4^. It was shown that COVID-19 dampened the circadian variation in core body temperature, circulating leukocytes and neutrophils, shifted the circadian variation in heart rate and caused uraemia^5^.

The new disease has generated a great deal of interest in the assessment of systemic immune status, but existing knowledge about mucosal immunity is clearly insufficient to understand the full pathogenetic mechanisms of the disease. The role of antibodies in neutralizing SARS-CoV-2 and offering protection from reinfection is well established. Humoral immunity against SARS-CoV-2 has been evaluated mainly at the system level in patients with a history of novel coronavirus infection (COVID-19) both with acute disease and in the convalescent phase^6–9^. Currently available serology tests assess immunoglobulin M (IgM) and IgG antibodies against the spike (S) and nucleocapsid (N) proteins of SARS-CoV-2. However, protective antibody levels are still under investigation^10,11^. The appearance of antibodies is paralleled by a gradual reduction in viral load, but serum antibodies can neutralize the virus in the blood, which is not sufficient for pathogen elimination^12^. The humoral mucosal immune response plays a crucial role in fighting COVID-19^13–15^.

SARS-CoV-2 primarily affects the upper respiratory tract. The immune response is initiated in the nasal and oropharyngeal mucosa, where nonspecific factors of innate immunity fight pathogens, particularly SARS-CoV-2. The immune response at the mucosal level is implemented through a single structured and highly specialized system, mucosa-associated lymphoid tissue (MALT), which consists of epithelial cells, lymphoid structures and submucosal immune molecules. The main components of mucosal immunity include saprophytic microflora, the epithelial cell layer, keratinization and salivary processes, antimicrobial peptides (lysozyme, defensins, lactoferrin, interferons, properdin), the complement system, lymphoid cells of innate immunity, natural killer cells, sIgA, IgG production and other factors^16,17^. The adaptive arm of mucosal immunity contributes greatly to viral eradication, but these processes have rarely been studied^18^.

Secretory immunoglobulin A is the main antibody class found on mucosal surfaces and produced by local plasma cells, primarily as an IgA dimer. It confers protection against various pathogens, including viruses, which is mediated by its neutralizing properties as well as its ability to prevent adhesion of pathogens to the mucosal surfaces and to opsonize pathogenic microorganisms, thus hindering their penetration into epithelial cells^19^. Preliminary studies have demonstrated high secretory levels of specific neutralizing sIgA in adults with acute COVID-19^15,20–22^. Immunoglobulin G is also found on the mucosal surfaces of the upper respiratory tract and is mainly derived from the blood circulation by passive leakage in the area of the gingival crevicular epithelium, but some is produced locally^23^. This class of immunoglobulin is known to have a high affinity, play a certain role in the recruitment of phagocytes and natural killer cells, activate the complement system, block the active centres of pathogens, and neutralize toxins^24^.

The absence of SARS-CoV-2 in the post-COVID-19 period does not necessarily mean complete recovery^25^. In recent studies among convalescent COVID-19 patients, a wide variety of persistent systemic symptoms have been reported, which has resulted in the introduction of new terms such as post-COVID-19 syndrome, long COVID-19, and chronic COVID-19. In October 2021, the World Health Organization (WHO) proposed a consensus definition for what they referred to as the “post-COVID-19 condition”. The condition was defined as the presence of symptoms lasting for at least 2 months in individuals with a history of probable or confirmed SARS-CoV-2 infection^26^. According to the WHO, this condition usually manifests 3 months from the onset of acute illness but cannot be explained by an alternative diagnosis. It is now clear that long COVID-19 has become a meaningful public health concern, given that it now affects millions of people worldwide^27^. Is there a correlation between immunological and clinical parameters in the post-COVID-19 period? Can shifts in humoral immunity be regarded as a sign or a component of post-COVID-19 syndrome? To date, there have been few studies on changes in the mucosal immune response, particularly the protective role of sIgA and IgG in subjects with a history of COVID-19.

The aim of this study was to evaluate the effects of novel coronavirus infection on the humoral factors of mucosal immunity in health care workers. The study objectives included an assessment of clinical condition using quality-of-life and fatigue questionnaires; an evaluation of the levels of total sIgA and IgG in the respiratory tract mucosa against the severity of the prior disease; and the investigation of a correlation between sIgA and IgG levels in different compartments of the upper and middle respiratory tract and between sIgA and IgG levels and clinical signs of post-COVID-19 syndrome.

## Materials

### Study design

Participation in the study was offered to 220 medical workers of the D.D. Pletnev hospital, Moscow, who ranged in age from 18 to 65 years who had had COVID-19 between April 2020 and May 2021 and those with no history of this infection. Voluntary informed consent to participate was signed by 202 subjects, thus the response rate was 91.8%. The exclusion criteria were recorded in 22 subjects, the main ones were: taking immunomodulatory or antiviral drugs within the last six months prior to the start of the study (n = 12 (54.5%)), immunization with any vaccine within 30 days prior to enrolment (n = 7 (31.8%)), presence of autoimmune disorder (n = 3 (13.6%)) or severe chronic diseases (n = 2 (9.1%)).

**Figure 1:**
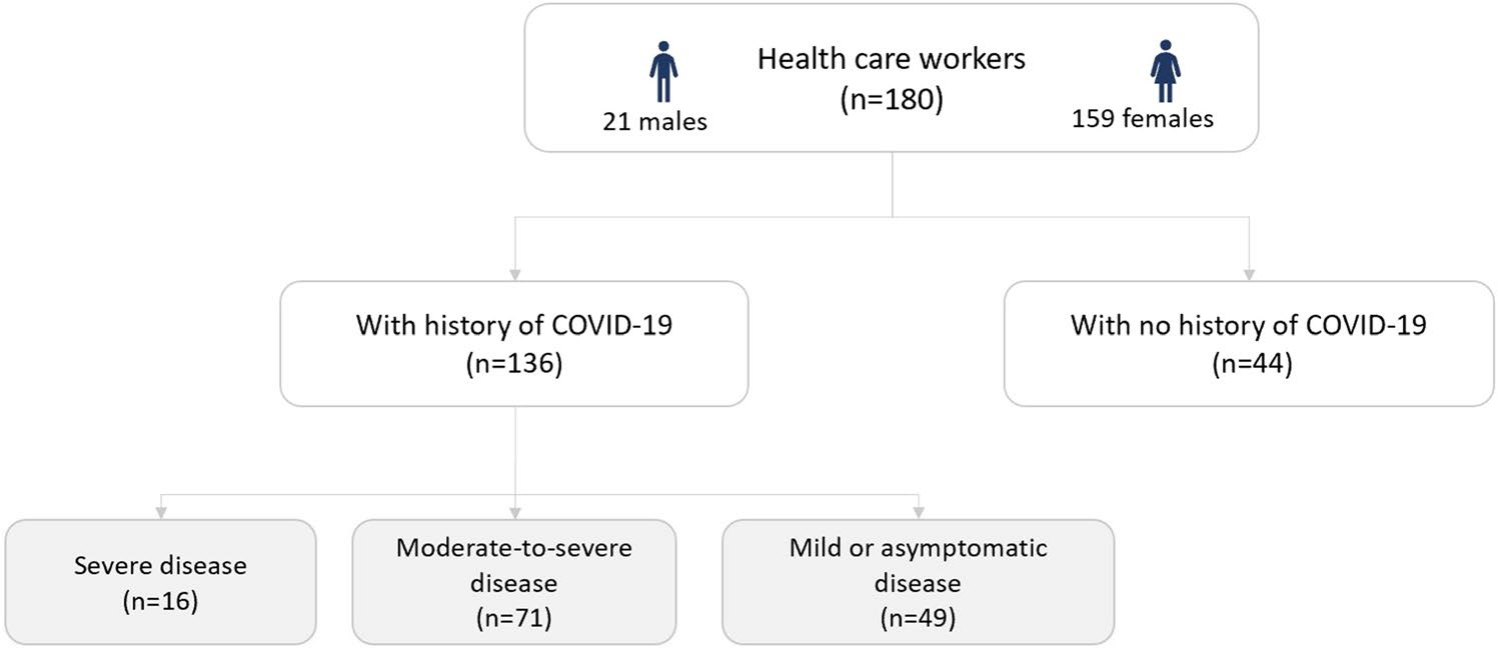
Flowchart of the distribution of participants into groups.

Therefore 180 health care workers (21 males and 159 females) were included in this one-stage, cross-sectional study 125.1 ± 71.1 days after disease onset. The Alpha (B.1.1.7), Beta (B.1.351) and Delta (B.1.617.2) variants of the virus prevailed in Russia at the specified time^28,29^. One hundred thirty-six subjects with a history of COVID-19 were divided into three groups depending on the severity of the prior^30^ disease: (1) severe disease (n = 16); (2) moderate-to-severe disease (n = 71); and (3) mild or asymptomatic disease (n = 49). The control group consisted of 44 medical workers who had no history of COVID-19 (Fig. 1).

This study included two visits but did not have a follow-up period.

At Visit 0 (Day 0), all subjects voluntarily signed the informed consent form, which met the requirements of the World Medical Association Declaration of Helsinki (2013), the Universal Declaration on Bioethics and Human Rights adopted on October 19, 2005, and the Universal Declaration of Human Rights adopted by the United Nations General Assembly on December 10, 1948. The study protocol was approved by the local ethics committee at Pirogov Russian National Research Medical University (Pirogov Medical University). All research was performed in accordance with relevant guidelines/regulations.

At Visit 1 (Day 1), clinical assessments were performed, including a detailed medical history and complaints and physical examination. The study subjects completed the SF-36 quality-of-life questionnaire and the FAS questionnaire. The Charlson comorbidity index was used to assess all the subjects. Salivary samples and nasal and oral mucosal epithelial scrapings were obtained. Samples of induced sputum were collected after inhalation of 5% hypertonic saline (fixed concentration) for up to 30 min in accordance with the standardized procedure^31^.

### Inclusion criteria

The following inclusion criteria were applied: age 18–65 years; history or no history of COVID-19; and voluntary signed written informed consent form.

### Exclusion criteria

The exclusion criteria were refusal to participate in this study; severe birth defects or severe chronic diseases; a history of cancer; a positive HIV or hepatitis B or C test in anamnesis; use of immunoglobulin or blood transfusion within the last three months prior to the start of the study; prolonged use (more than 14 days) of immunosuppressive, immunomodulatory or antiviral drugs within the last six months prior to the start of the study; any surgery or oral inflammation within one month prior to enrolment; any known or suspected immunosuppressive, immunodeficiency or autoimmune disorder; chronic alcohol abuse and/or a history of drug use; immunization with any vaccine within 30 days prior to enrolment; prior immunization with an experimental or approved SARS-CoV-2 vaccine; resolved acute infection within less than a month prior to the start of the study; pregnancy or lactation; simultaneous participation in another clinical study; and inability to comply with the study protocol requirements.

## Methods

### Clinical methods

Clinical assessments were performed, including a detailed medical history and complaints and physical examination and analysis of chest computed tomography data. The primary data from the SF-36 questionnaire were interpreted using a predefined method^32^, i.e., an assessment of direct items, where higher scores indicated a greater influence of the item on quality of life (physical functioning, vitality, general health, social functioning, and mental health), and reverse items, where higher scores indicated a lower influence of the item on quality of life (physical role functioning, emotional role functioning, and bodily pain). Interpretation of the FAS data included an assessment of physical and mental scores as well as the total score, with values of the total score > 22 indicating substantial fatigue^33^.

### Laboratory tests

Laboratory studies were carried out as samples were received from June 19, 2020, to October 22, 2021. Levels of immunoglobulins in mucosal secretions were measured by enzyme-linked immunosorbent assay (IgA secretory-EIA-BEST and IgG total-EIA-BEST, Vector Best, Novosibirsk, Russian Federation) based on a two-step sandwich enzyme immunoassay using monoclonal antibodies (mAbs) against IgG and the secretory component linked to the alpha chain of IgA. The tests were performed strictly in accordance with the manufacturer's recommended protocol.

#### Determination of sIgA

Standards with known concentrations of sIgA and the samples were added to the wells of a plate coated with an anti-sIgA mAb. The plate was then incubated according to the test kit instructions. The intensity of the developed colour was proportional to the concentration of sIgA in the sample. The concentration of sIgA was calculated using the standard curve and measured optical density values.

#### Determination of IgG

First, standards with known concentrations of total IgG and samples were added to the wells of a strip plate coated with an anti-human IgG gamma chain mAb and then incubated. Next, the bound IgG in the plate wells was treated with a conjugate of peroxidase and anti-human IgG lambda and kappa light chain mAbs.

The resulting immune complexes consisting of immobilized mAbs, IgG, and the conjugate were detected by an enzymatic reaction with tetramethylbenzidine as a substrate. The intensity of the developed colour was proportional to the concentration of total IgG in the sample. The concentration of total IgG was calculated using the standard curve and measured optical density values.

#### Determination of specific anti-SARS-CoV-2 antibodies

Quantitative determination of IgG antibodies to SARS-CoV-2 was based on a two-step sandwich immunoassay (SARS-CoV-2 IgG (CLIA), Shenzhen Mindray Bio-Medical Electronics Co., Ltd., China).

First, serum specimens were incubated with paramagnetic particles coated with a SARS-CoV-2 antigen. A monoclonal mouse anti-human IgG alkaline phosphatase conjugate in MES buffer was then added to the reaction vessel. After incubation, the conjugate became bound to the anti-SARS-CoV-2 IgG antibodies captured on the paramagnetic particles. Finally, the chemiluminescent signal was measured and expressed in relative light units (RLUs). The chemiluminescence intensity in RLUs was proportional to the number of anti-SARS-CoV-2 IgG antibodies in the sample. The concentration of anti-SARS-CoV-2 IgG antibodies was determined against the standard curve.

### Statistical analysis

Statistical analysis was performed by parametric and nonparametric methods using IBM Corp. Released 2015. IBM SPSS Statistics for Windows, Version 23.0. Armonk, NY: IBM Corp. software.

Descriptive statistics were used to analyse numerical variables. The normality of the distribution of variables was determined using the Shapiro–Wilk test. Parametric criteria were used for normally distributed variables. In this case, the significance of differences in quantitative parameters between the groups was determined using the unpaired Student’s t test. In the absence of a normal distribution of variables, the significance of differences was assessed using the nonparametric Mann–Whitney U test. Depending on the type of distribution, the following measures of central tendency and dispersion were computed: means and root mean square (standard) deviations (M ± SDs) or medians and 25th and 75th percentiles (Me [Q1; Q3]). The differences were considered statistically significant at *p* < 0.05. The correlation between two parameters was evaluated by Spearman correlation analysis.

## Results

### Clinical characteristics of the study groups

This study was conducted 125.1 ± 75.0 days following the onset of the first symptoms. The health care workers with a history of severe or moderate-to-severe novel coronavirus infection were older and had a higher body mass index (BMI) and Charlson comorbidity index than subjects with a history of mild or asymptomatic disease and those with no history of COVID-19 (Table 1).

**Table 1:** Clinical characteristics of the study groups.

Group Parameter	Age M ± SD	BMI M ± SD	Charlson Comorbidity Index Me (Q1; Q3)
Severe disease	55.1 ± 6.3**	32.4 ± 3.8**	2 (1;3)*
Moderate-to-severe disease	52.2 ± 10.2**	29.2 ± 6.1	1 (2;1)*
Mild/asymptomatic disease	39.5 ± 13.2	27.6 ± 6.8	0 (0;1)
No history of COVID-19	44.9 ± 13.2	28.2 ± 6.5	1 (0;2)

*p < 0.05 compared to subjects with a history of mild/asymptomatic disease and those with no history of COVID-19

**p < 0.005 compared to the subjects with a history of mild/asymptomatic disease and those with no history of COVID-19.

Although this study was conducted long after recovery, analysis of the data collected from the quality-of-life SF-36 questionnaire showed that all the subjects with a history of COVID-19 reported health problems that limited their daily activities and changes in their emotional health that affected their functioning, regardless of disease severity. We identified the presence of physical, mental and cognitive signs of long-term COVID in HCWs. Compared to the subjects who had mild or asymptomatic COVID-19 and subjects with no history of COVID-19, the subjects with a history of moderate or moderate-to-severe infection had substantial changes in general health, vitality, and social functioning parameters. The disease did not have a substantial impact on the subjects’ mental health and did not affect their feelings of calmness or complacency.

An assessment of the FAS data showed that the subjects with a history of COVID-19 had higher total fatigue scores, mainly because of the physical fatigue score (Table 2).

**Table 2:** Assessment of the quality of life (SF-36 questionnaire) and fatigue (FAS) parameters.

Group Parameter	Severe disease	Moderate-to-severe disease	Mild/asymptomatic disease	No history of COVID-19
QoL (SF-36) assessment Me (Q1; Q3)				
Physical functioning	45.0 (25.0; 80.0)*	60.0 (45.0; 100.0)	95.0 (80.0; 95.0)	92.5 (83.6; 100.0)
Physical role functioning	25,0 (0.0; 62.5)*	75.0 (25.0; 100.0)*	75.0 (50.0; 100.0)*	100.0 (100.0; 100.0)
Bodily pain	70.0 (46.5; 87.0)	100.0 (41.0; 100.0)	94.0 (68.0; 100.0)	100.0 (100.0; 100.0)
General health	72.0 (53.5; 75.0)	55.0 (50.0; 67.0)*	72.0 (58.5; 82.0)	77.0 (65.8; 87.0)
Vitality	40.0 (17.5; 47.5)*	45.0 (35.0; 85.0)	50.0 (40.0; 70.0)	67.5 (53.8; 75.0)
Social functioning	100.0 (37.5; 100.0)	62.5 (50.0; 82.5)*	87.5 (62.5; 100.0)	100 (87.5; 100.0)
Emotional role functioning	33.3 (16.7; 100.0)*	66.7 (33.3; 100.0)*	66.7 (33.3; 100.0)*	100.0 (66.7; 100.0)
Mental health	52.0 (48.0; 78.0)	60.0 (48.0; 80.0)	64.0 (42.0; 78.0)	72.0 (58.0; 80.0)
Fatigue Assessment Scale (FAS) Me (Q1; Q3)				
Total FAS score	31.0 (20.0; 35)*	17.5 (15.3; 21.8)*	23.0 (17.0; 27.0)*	19.0 (16.0; 21.0)
Mental fatigue score (FAS)	12.0 (8.5; 14.5)	8.0 (6.3; 9.0)	10.0 (7.0; 12.0)	8.0 (7.0; 9.0)
Physical fatigue score (FAS)	19.0 (11.5; 20.0)*	10.5 (8.0; 13.8)	13.0 (9.0; 16.0)	11.0 (9.0; 12.0)

*p < 0.05 compared to the control group.

### Laboratory test data

**Figure 2:**
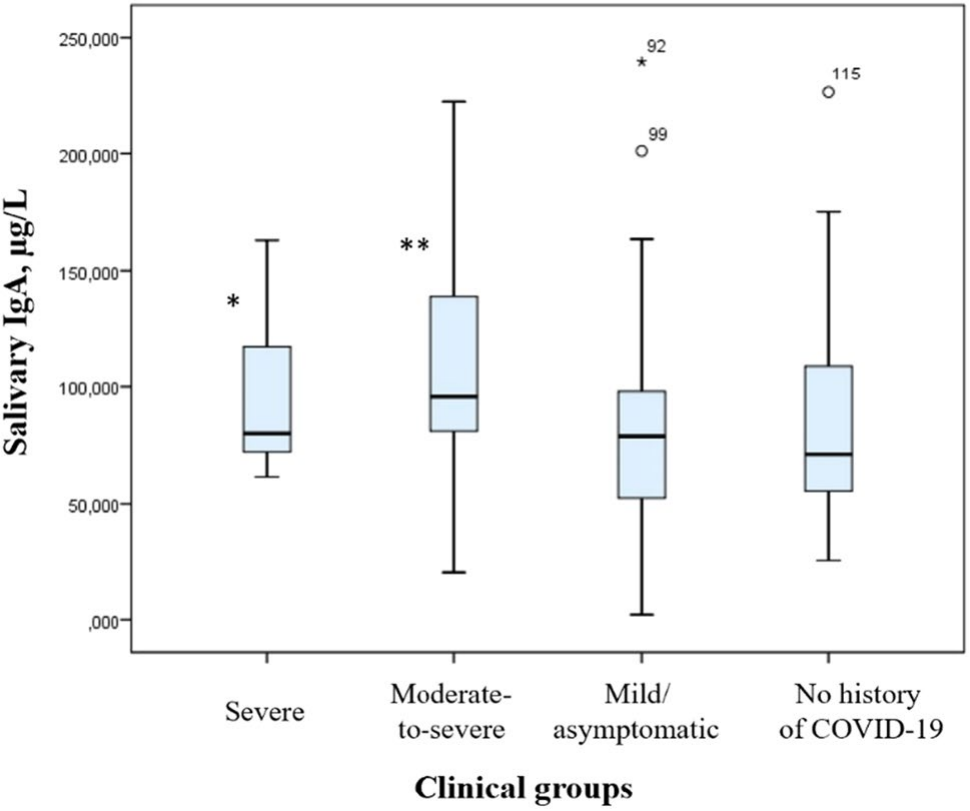
Concentration of sIgA in salivary gland secretions, µg/L. Note: *p < 0.05 compared to the control group, **p < 0.005 compared to the control group.

Concentrations of sIgA in different mucosal compartments and secretions are shown in Table 3. Among subjects who had severe or moderate-to-severe COVID-19, salivary sIgA levels were significantly higher than those in the control group (Fig. 2).

**Table 3:** Comparison of sIgA levels in secretions and different mucosal compartments by severity of prior disease, µg/L.

Group sample type	Severe disease	Moderate-to-severe disease	Mild/asymptomatic disease	No history of COVID-19
Oropharynx	20.5 (15.1; 28.9)**	12.4 (2.6; 23.5)	8.9 (2.3; 22.6)	6.5 (1.1; 14.2)
Nasopharynx	40.4 (18.3; 70.9)	33.0 (19.8; 56.0)	48.8 (21.4; 58.2)	29.9 (19.2; 58.2)
Induced sputum	73.2 (23.3; 85.5)	64.5 (37.8; 86.7)	45.0 (11.2; 83.2)	42.9 (13.1; 69.0)
Salivary gland secretions	80.0 (71.6; 121.8)*	95.8 (78.4; 139.3)**	78.8 (49.8; 99.7)	71.1 (54.2; 111.9)

Me (Q1; Q3) *p < 0.05 compared to the control group

**p < 0.005 compared to the control group.

Compared to the control group, these subjects also had higher sIgA levels in induced sputum samples and samples collected from the oropharynx, but this elevation was not statistically significant.

A tendency towards correlation was observed between the levels of sIgA in the oropharynx and nasopharynx (r = 0.171, *p* < 0.05) and between the levels of sIgA in the oropharynx and salivary gland secretions (r = 0.226, *p* < 0.05). A direct interrelation was detected between sIgA levels in induced sputum and salivary gland secretions (r = 0.375, *p* < 0.01). An inverse correlation was demonstrated between the levels of total sIgA in all mucosal compartments and the number of days between the onset of disease and the study period (r = 0.278, *p* < 0.05).

**Figure 3:**
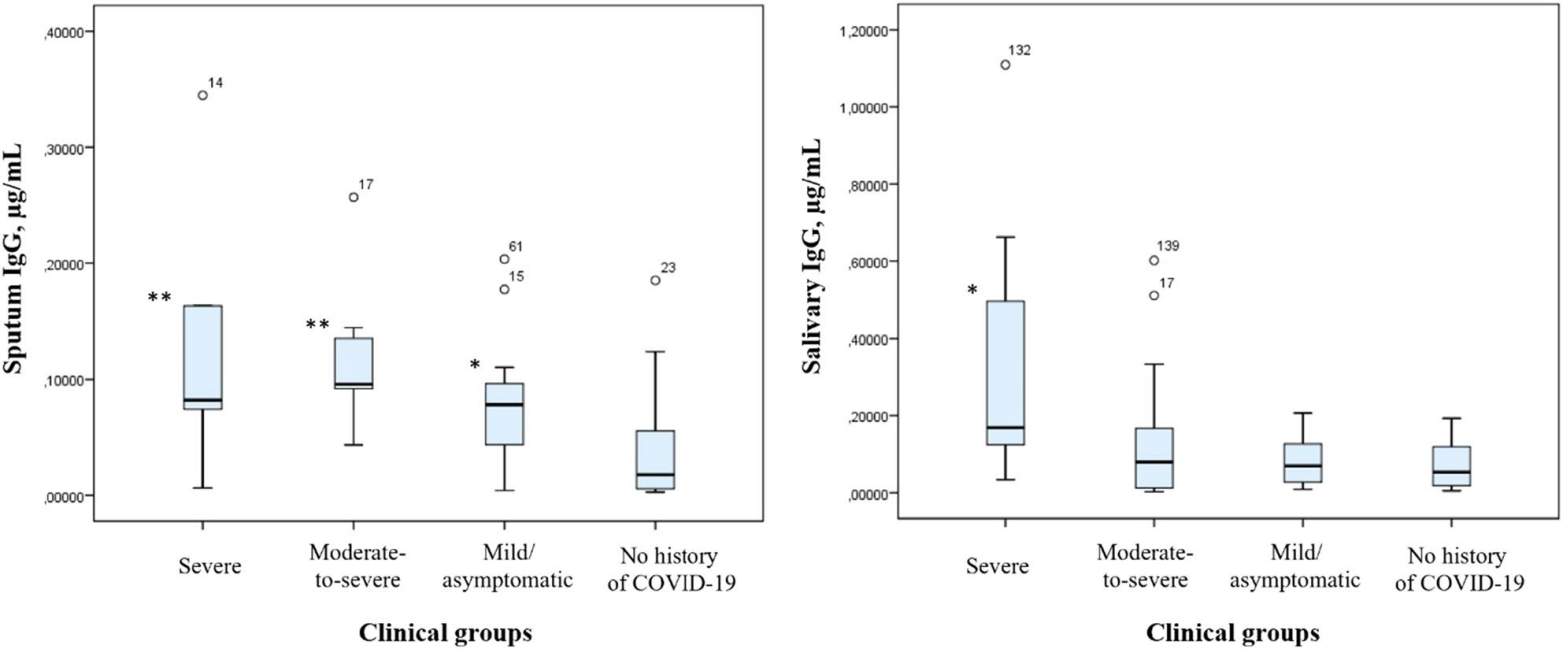
Concentration of total IgG in salivary gland and bronchial secretions, µg/mL. Note: *p < 0.05 compared to the control group, **p < 0.005 compared to the control group.

The concentrations of total IgG in various samples are shown in Table 4. Compared to the subjects in the control group, all the subjects with prior COVID-19 had significantly higher levels of total IgG in induced sputum. In the group of patients who had had severe infection, total IgG levels were also significantly higher in saliva (Fig. 3). In contrast, the subjects with a history of severe or moderate-to-severe COVID-19 had lower levels of total IgG in the nasopharynx than those with a history of mild or asymptomatic disease and those who had not had this infection.

**Table 4:** Comparison of total IgG levels in secretions and different mucosal compartments by severity of prior disease, µg/mL.

Group sample type	Severe disease	Moderate-to-severe disease	Mild/asymptomatic disease	No history of COVID-19
Oropharynx	0.012 (0.008; 0.101)	0.014 (0.009; 0.0154)	0.009 (0.007; 0.068)	0.008 (0.007; 0.034)
Nasopharynx	0.040 (0.018; 0.259)*	0.023 (0.009; 0.084)**	0.208 (0.047; 0.366)	0.115 (0.027; 0.175)
Induced sputum	0.082 (0.057; 0.209)**	0.096 (0.069; 0.139)**	0.078 (0.042; 0.097) *	0.018 (0.005; 0.057)
Salivary gland secretions	0.182 (0.107; 0.640)*	0.079 (0.012; 0.168)	0.070 (0.025; 0.135)	0.053 (0.018; 0.1240)

Me (Q1; Q3) *p < 0.05 compared to the control group

**p < 0.005 compared to the control group.

Comparison of the key quality-of-life and humoral immunity parameters revealed a correlation between total IgG levels, on the one hand, and some quality-of-life parameters, as assessed by the SF-36 questionnaire, and FAS scores (level of fatigue), on the other hand (Table 5).

**Table 5:** Correlation coefficients between IgG levels and some quality-of-life parameters (SF-36) and fatigue scores (FAS).

Location parameter	IgG levels in oropharynx	IgG levels in nasopharynx	IgG levels in saliva
Physical functioning	−0.407*	–	−0.514**
Social functioning	–	0.607**	–
Mental health	–	0.536**	–
Total FAS score	−0.436*	–	–
Physical fatigue score (FAS)	−0.548**	–	–

*Significant correlation at the 0.05 level (bilateral correlation)

**Significant correlation at the 0.01 level (bilateral correlation).

Analysis of the relationship between sIgA and IgG demonstrated a direct interrelation between these parameters in the same mucosal compartments and in matched samples of glandular secretions from the upper and middle respiratory tract. A direct correlation was also detected between the levels of total IgG in all mucosal compartments and the serum levels of specific IgG antibodies against SARS-CoV-2 (Table 6). In contrast to sIgA, there was no relationship detected between the IgG levels in various mucosal compartments and the period between the time of disease resolution and the study start. This may be explained by a gradual increase in IgG levels over time after the onset of the disease and their gradual decrease within three months^34^.

**Table 6:** Correlation coefficients (r) between total IgG levels and sIgA levels in matched samples and between total IgG levels and serum levels of specific anti-SARS-CoV-2 IgG antibodies.

**r**	IgG levels in oropharynx	IgG levels in nasopharynx	IgG levels in induced sputum	IgG levels in saliva
sIgA (matched samples)	0.301**	0.336**	0.340*	0.316**
anti-SARS-CoV-2 IgG antibodies	0.320*	0.296*	0.475**	0.390**

*Significant correlation at the 0.05 level

**Significant correlation at the 0.01 level.

## Discussion

Our study population included HCWs who were at high risk of contracting novel coronavirus infection, becoming asymptomatic carriers and therefore passing the virus on to patients or colleagues. Although the mortality rate among HCWs is lower (0.5% proportion of fatal cases^35^) than the rate of mortality in the general population reported in the literature, better overall health and care among HCWs, different age demographics, and other factors could explain these differences. However, due to exposure to numerous infected individuals, HCWs, if infected, could be characterized by a higher viral load, which is associated with worse clinical outcomes^36^.

The major risk factors for a severe clinical course and outcomes of COVID-19 have been identified as advanced age, male sex, ethnicity, and preexisting hypertension, diabetes, obesity, chronic obstructive pulmonary disease, interstitial lung disease, tumours, immunodeficiencies and other conditions^37^. HCWs with a history of severe or moderate-to-severe infection were older and had a higher BMI and comorbidity index, which is consistent with data about the main risk factors for severe COVID-19^30^.

Long COVID is a multisystem disease that develops regardless of initial disease severity. Its clinical spectrum comprises a wide range of symptoms^38^. Although this study was conducted approximately three months after the onset of disease, changes in quality-of-life parameters and FAS scores were still present. An assessment of the SF-36 data from the groups of patients with a history of severe or moderate-to-severe infection showed that the most significant changes were seen in the physical functioning, vitality, and social functioning domains. Unlike in the control group, all health care workers with a history of COVID-19 reported emotional problems that limited their daily activities, regardless of disease severity. Fatigue, in particular physical fatigue, was reported only by subjects with a history of mild or severe COVID-19.

Our data characterized post-COVID-19 syndrome, which had still not fully resolved three or more months after the onset of infection. Some studies have shown that 50% to 80% of convalescent COVID-19 patients report having nonspecific symptoms, most often fatigue, headache, dyspnoea, anosmia, and memory impairment^38,39^. A systematic review and meta-analysis reported more than 50 potential long-term effects of SARS-CoV-2 infection^40^. In their study, Carfì et al. showed that only 12.6% of the patients completely recovered; 55% of the patients had three or more symptoms, and 44% reported decreased quality of life 60 days post-onset of the first COVID-19 symptoms^41^.

The lack of studies on the humoral arm of mucosal immunity in the post-COVID-19 period stimulated our interest in evaluating sIgA and IgG levels in different mucosal compartments of the respiratory tract. Ren C. et al. showed that sIgA in respiratory mucosa could be detected on the first day after illness onset, and the median conversion time for sIgA in sputum, throat swabs, and serum was 3, 4, and 10 days, respectively. During the recovery period, sIgA-positive rates in sputum and throat swabs gradually decreased from 60.7% and 57.1% 1 month after illness onset, respectively, and sIgA antibodies were all undetectable after 6 months^42^.

Our study focused on the measurement of total sIgA levels and showed that total salivary sIgA levels were higher in subjects with a history of severe or moderate-to-severe COVID-19 than those in the control group. In addition, a trend towards elevation of these antibodies in oropharyngeal and induced sputum samples was detected for the same groups of patients, but it was not statistically significant.

These changes could possibly be explained by persistence of inflammatory reactions on the mucous membranes of the respiratory tract and the presence of some specific anti-SARS-CoV-2 immunoglobulins. Levels of anti-SARS-CoV-2 sIgA have been measured in a number of studies. For example, Isho B. et al. reported that in COVID-19 patients, salivary sIgA antibody responses to the receptor-binding domain (RBD) of the S protein measured long after recovery (up to 115 days) were higher than those in the control group^34^. As in our study, secretory IgA antibodies in salivary gland secretions decayed over time. Moreover, the authors did not find a strong correlation between IgA levels in the blood and saliva, which suggested that the IgA response in the oral cavity differs from that in peripheral blood.

We found that, unlike sIgA, total IgG was elevated not only in saliva but also in samples of induced sputum from patients who had severe or moderate-to-severe COVID-19. This is consistent with the data reported by Isho B. et al., who showed that anti-SARS-CoV-2 IgG levels were strongly correlated across paired serum and saliva samples, indicating that saliva could be used for monitoring the immune response to SARS-CoV-2 infection^34^. Randad et al. also evaluated the correlation of results obtained from saliva vs. serum and determined the sensitivity and specificity for each diagnostic medium, stratified by antibody isotype, for the detection of SARS-CoV-2 infection based on COVID-19 case designation for all specimens; matched serum and saliva SARS-CoV-2 antigen-specific IgG responses were significantly correlated^43^.

Levels of total IgG correlated with those of sIgA in the same mucosal compartments, anti-SARS-CoV-2 IgG, and some parameters of general health. Levels of IgG in secretions and mucosal scrapings from the upper respiratory tract did not correlate with the number of days post-disease onset. However, we did not measure titres of specific mucosal antibodies.

Interestingly, subjects with a history of severe COVID-19 had lower titres of IgG in the nasopharynx. The study by Alkharaan H. et al. showed that salivary IgG antibody responses in patients with a history of mild COVID-19 were detectable up to 9 months after recovery, with high correlations between spike and nucleocapsid specificity. At 9 months, IgG remained in the blood and saliva of most patients. Salivary IgA was rarely detected at this time point. Scientists have also demonstrated the tolerance of salivary IgG to temperature and detergent pretreatments^44^. Upon recovery, asymptomatic patients exhibited sustained salivary IgG antibodies against SARS-CoV-2^45^. The nasal mucosa is the entry point for SARS-CoV-2. Our study demonstrated that subjects who had mild or asymptomatic disease had significantly (twofold) higher levels of immunoglobulins, which is evidence for strong protection from reinfection. Subjects who recovered from severe or moderate-to-severe COVID-19 had three- or fivefold lower levels of the studied parameter, which may be a sign of immunodeficiency associated with damage to epithelial cells by the virus, as well as, probably, age-related atrophy of the nasal mucosa epithelium, given the predominance of the older category of persons in these groups. The obtained results are not described in the literature and require further study.

Our study has a number of limitations. First, it was a one-stage study, which means that we could not follow up on the changes in clinical signs or levels of mucosal immunoglobulins. Nevertheless, the study results suggest the presence of long-term changes in post-COVID-19 syndrome. Second, the absence of strain detection among our participants is also a limitation of our study. Finally, we measured only total sIgA, but it is highly likely that its elevation is a sign of a specific anti-SARS-CoV-2 immune response in the respiratory mucosa. Therefore, this opens up new possibilities for using these laboratory tests to assess the level of mucosal immunity without the need for costly methods. The same is true for evaluating total IgG in different mucosal compartments.

Quantification of salivary IgA and IgG antibodies can elucidate mucosal and systemic immune responses after natural infection in the post-COVID-19 period before or after vaccination. Noninvasive saliva-based SARS-CoV-2 antibody measurement may be a complementary alternative to conventional blood serology. The preliminary findings suggest that the measurement of salivary and serum IgG and IgA merits further investigation as markers of current or recent SARS-CoV-2 infection^46^ and may contribute to the understanding of post-COVID syndrome in the context of immune changes.

We investigated only a few parameters of mucosal immunity, it was found that individuals who had had a severe and moderate course of infection had elevated levels of total sIgA in saliva samples, as well as levels of total IgG in all areas of the mucous membranes of the respiratory tract, with the exception of the nasal cavity. Elevated levels of immunoglobulins three months after the infection may indicate the persistence of inflammatory reactions, which also correlates with the manifestations of post-COVID syndrome and requires further study. Local immunodeficiency in the nasal cavity may indicate viral damage to epitheliocytes, which might lead to frequent respiratory infections, and probably requires additional immunocorrective approaches aimed at enhancing mucosal immunity, for example, nasal form of medication containing bacterial ligands or recombinant interferons.

## Conclusion

Although the upper respiratory tract mucosa is where the virus replicates and the immune response is initiated, there are few publications on anti-COVID-19 mucosal immunity, its impact on the course of the disease, and its status in the post-COVID-19 period. Our study demonstrated long-term changes in the humoral mucosal immune response, which were most pronounced in health care workers with a history of severe or moderate-to-severe COVID-19, and an association of these changes with certain clinical characteristics of health care workers with post-COVID-19 syndrome. Studying the humoral immunity of the respiratory mucosa is likely to improve the understanding of the immune response to this emerging disease, help contain the pandemic develop approaches for immunorehabilitation of persons who have had COVID-19 and effective vaccines aimed at protecting the mucous membranes as a major gateway for viral infections.

## Data Availability

The datasets generated and/or analysed during the current study are available from the corresponding author upon reasonable request.
